# Clinical vitamin D levels are associated with insular volume and inferior temporal gyrus white matter surface area in community-dwelling individuals with knee pain

**DOI:** 10.3389/fnins.2022.882322

**Published:** 2022-08-31

**Authors:** Larissa J. Strath, Pedro Valdes Hernandez, Chavier Laffitte Nodarse, Alisa J. Johnson, Jeffrey D. Edberg, Roger B. Fillingim, Yenisel Cruz-Almeida

**Affiliations:** ^1^Pain Research and Intervention Center of Excellence, University of Florida, Gainesville, FL, United States; ^2^Department of Community Dentistry and Behavioral Science, University of Florida, Gainesville, FL, United States; ^3^School of Medicine, The University of Alabama at Birmingham, Birmingham, AL, United States

**Keywords:** vitamin D, insula, white matter, brain, chronic pain

## Abstract

**Context:**

Vitamin D is an essential, fat soluble micronutrient long-known for its effects on calcium homeostasis and bone health. With advances in technology, it is being discovered that Vitamin D exerts its effects beyond the musculoskeletal system. Vitamin D has since been noted in nervous system health and functioning, and is becoming a target of interest in brain health, aging, and chronic pain outcomes.

**Objectives:**

We and others have previously shown that deficient Vitamin D status is associated with greater pain severity across a variety of conditions, however the reason as to why this relationship exists is still being understood. Here, we sought to examine associations between Vitamin D status and brain structure in those with chronic knee pain.

**Methods:**

Structural MRI imaging techniques and whole brain analyses were employed and serum Vitamin D were collected on 140 participants with chronic pain. Covariates included age, sex, race and site, as these data were collected at two separate institutions. ANOVAs using the clinical cut points for Vitamin D status (deficient, insufficient, and optimal) as well as continuous regression-based Vitamin D effects were employed to observe differences in brain volume. *P*-value was set to 0.017 after correction for multiple comparisons.

**Results:**

We discovered that individuals in our sample (age = 50+; 63.6% female; 52.1% Non-Hispanic Black) who were either clinically deficient (<20 ng/mL) or insufficient (20–30 ng/mL) in serum Vitamin D had significant differences in the gray matter of the left circular insular cortex, left inferior temporal gyrus, right middle temporal gyrus, as well as decreased white matter surface area in the right inferior temporal gyrus compared to those considered to have optimal levels (>30 ng/mL) of serum Vitamin D.

**Conclusion:**

Evidence from these data suggests that Vitamin D, or lack thereof, may be associated with pain outcomes by mediating changes in regions of the brain known to process and interpret pain. More research understanding this phenomenon as well as the effects of Vitamin D supplementation is warranted.

## Introduction

Vitamin D (cholecalciferol) is a secosteroid that is essential for optimal health and functioning. Historically, it has been known for its role in calcium homeostasis and bone structure after it was discovered to be a missing nutrient in children with Rickets – a disease involving severe bone demineralization (Ward et al., 2007). While many commonly associate Vitamin D with its skeletal importance, it has been discovered that Vitamin D also has many other functions in the body, crossing systems and mechanisms alike. The discovery of the Vitamin D receptor (VDR) and its presence on many different types of cells has opened the door to a new understanding of the influence Vitamin D on health outcomes (Pike and Meyer, 2012). The VDR is present on many types of cells and target organs, implicating this nutrient in cell differentiation and proliferation (Berridge, 2015), and accelerated epigenetic aging (Vetter et al., 2020) among others. Additionally, Vitamin D is necessary for energy production and mood stability, as noted by excessive tiredness, fatigue and depression in those deficient in it (Holick, 2015). Clinical studies also suggest that Vitamin D deficiency may be involved in diseases of the nervous system (Wrzosek et al., 2013), cardiovascular system (Danik and Manson, 2012), and endocrine system (Slovik, 1983).

Recently, Vitamin D has been implicated in the pain experience by potentially altering mechanisms by which pain is sensed and modulated. Previous research has demonstrated that Vitamin D levels are often lower in individuals with chronic pain compared to those without (Martin and Reid, 2017). We have also previously documented greater pain severity in individuals with knee osteoarthritis (KOA) who were deficient in Vitamin D (Glover et al., 2012). There is some evidence that suggest how Vitamin D could modulate the pain experience peripherally. For example, it has been documented that optimal Vitamin D status can enhance the effects of monocytes and macrophages in the immune system and lead to decreased levels of inflammation (Zhang et al., 2012). Immune system disruption and inflammation have been implicated in the development and maintenance of pain, as pro-inflammatory cytokines and chemokines can bind to and activate nociceptors (i.e., pain-sensing neurons) (Totsch and Sorge, 2017). Additionally, Vitamin D/VDR has been documented to interact with specific pain signaling pathways in the dorsal root ganglion including nerve growth factor (NGF), glial-derived neurotrophic factor (GDNF), epidermal growth factor receptor (EGFR) and various types of opioid receptors (Habib et al., 2020). As one can see, due to the complex nature of pain, the potential effects of Vitamin D on pain outcomes extend beyond the periphery and into the central nervous system.

Brain structures involved in the sensing, processing, modulation and reaction to pain signals include the primary and secondary somatosensory cortices, anterior cingulate cortex (ACC), prefrontal cortex (PFC), insular cortex, amygdala, thalamus, cerebellum and periaqueductal grey matter (PAG) (Yang and Chang, 2019). Pain has also been associated with changes in white matter integrity – tracts of neurons that connect and allow communication between various brain regions (Cruz-Almeida et al., 2017). It has been hypothesized that individuals with chronic pain conditions have differences in structure and function of these various brain regions compared to those without pain, potentially exacerbating the experience. Thus, the purpose of this investigation was to examine associations between Vitamin D status and the brain structures involved in pain processing in individuals in a chronic pain state, KOA.

## Materials and methods

### Participants

This is a secondary investigation from a study of middle-to-older aged adults (45–85 years old) with and without knee pain recruited from the University of Florida (UF; Gainesville, FL, United States) and the University of Alabama at Birmingham (UAB; Birmingham, AL, United States). Exclusion criteria included if they reported: (1) significant surgery to the most painful knee (e.g., total knee replacement); (2) presence or history of cardiovascular disease; (3) uncontrolled hypertension (blood pressure > 150/95 mmHg); (4) systemic rheumatic diseases (e.g., rheumatoid arthritis, systemic lupus erythematosus, and fibromyalgia); (5) neuropathy; (6) chronic opioid medication use; (7) serious psychiatric illness; (8) neurological disease (e.g., Parkinson’s, multiple sclerosis, stroke with loss of sensory or motor function, or uncontrolled seizures); (9) pregnant; (10) significantly greater pain in a body site other than the knee. All participants provided written informed consent and the study was IRB approved at both sites. Our participants were recruited as part of a parent study aimed to examine ethnic and race group differences in physical symptoms, psychosocial functioning, and pain-related central nervous system structure and functioning in knee osteoarthritis. After an initial phone screening, a Health Assessment Session (HAS) was scheduled to obtain informed consent prior to study procedures. During the HAS, a health history including depression, anxiety, and pain history that included movement-evoked pain intensity and interference, and physical exam were conducted. Chronic knee pain was defined as pain that has been present or recurrent for 6 months or longer. The present study is an ancillary investigation that aimed to determine associations between brain and vitamin D levels in those with knee pain, thus, only measures relevant to the study hypotheses are included and presented below. Full study procedures have been reported elsewhere(Fullwood et al., 2021). Univariate Analyses of Variance (ANOVAs) were used to assess demographic information and any differences in Body Mass Index (BMI) between the groups.

### Graded-chronic pain scale (G) measures of pain intensity and interference

The GCPS is a robust, validated (Elliott et al., 2000) self-reported questionnaire that measures two dimensions of chronic pain severity: pain intensity and pain-related disability. The questionnaire consists of seven items, with six scored on an 11-point Likert scale asking participants to report their current, average and worst pain over the last 6 months (i.e., 0 = “no pain” to 10 = “pain as bad as it can be”), and how much pain has interfered with daily activities, recreation/social/family activities, and ability to work (i.e., 0 = “no interference” to 10 = “unable to carry out activities”). Scores are then calculated for the two subscales: characteristic pain intensity is calculated as the mean intensity ratings for the current, worst and average pain multiplied by 10; and the pain-related disability score, which is calculated as the mean rating for difficult performing daily, social and work-related activities multiplied by 10, with each score ranging from 0 to 100. One open-ended question asks participants to report “how many days in the last 6 months have you been kept from your usual activities because of pain.” Higher scores indicate greater pain and pain-related disability.

### Vitamin D assay and clinical cutoff groups

Blood was drawn into a 7cc Corvac SST and wrapped in foil to protect from light. After 30 min, samples were centrifuged at 1800G for 10 min and then transferred to a 0.5 mL serum aliquot into an amber cryovial and stored at −80°C until processed for assays. Vitamin D was measured on a TOSOH Bioscience AIA-900 (South San Francisco, CA, United States) using immunofluorescence. To examine the main effect of Vitamin D, serum level in ng/mL was used as continuous data. To examine group effects, participants were grouped based on the current clinical cutoffs for serum Vitamin D: deficient (<19.99 ng/mL), insufficient (20.00 – 29.99 mg/mL) and optimal (>30.00 ng/mL). Due to the effect that adipose tissue can have on circulating vitamin D, a correlation analysis was employed to determine whether or not body mass index (BMI) was related to Vitamin D status.

### Brain imaging acquisition

MRI data was acquired for participants at the University of Florida using a 3.0 Tesla Philips Achieva whole body scanner with a 32-channel head coil, and at the University of Alabama, Birmingham with an 8-channel head coil. To minimize movement and preserve data quality, participant’s heads were secured *via* cushions positioned inside the head coil. T1-weighted (T1w) images were acquired using a high-resolution three-dimensional (3D) MP-RAGE sequence (repetition time = 7.0 ms, echo time = 3.2 ms/8°, 1 mm^3^ isotropic voxels).

### Brain imaging analysis

T1w images were processed using FreeSurfer 7.1.0’s *‘‘recon-all’’* function with default settings to calculate several vertex-wise morphometric measures of cortical area, cortical thickness, cortical volume, pial surface area, cortical sulcus (i.e., measures the depth/height of each vertex above the average mid cortical surface), smoothed cortical curvature and Jacobian white matter (a measure of the area of the surface defining the interface between gray and white matter determined by the amount of distortion needed to warp a subject into the standard normalized spherical space). We used QATools, a set of quality assurance/QC scripts for FreeSurfer processed structural MRI data,^1^ to check the quality of the FreeSurfer results. No participant had any topological defects in their cortical extracted surfaces.

Differences in each of these morphometric measures between Vitamin D cutoff groups were tested using Freesurfer’s “*mri_glmfit”* function, controlling for age, gender, ethnicity/race, and study site, making a one-way ANCOVA with 3 levels. Brain regions where these differences were significant were detected using Freesurfer’s Cluster-wise Correction for Multiple Comparisons to account for false positive rates, setting *p* < 0.01 (two-tailed) at the vertex level and CWP < 0.05 at the cluster level (Greve and Fischl, 2018)—CWP is the cluster wise *p*-value corrected using Bonferroni to account for both hemispheres (i.e., multiplied by 2). Moreover, *post-hoc* pair-wise comparisons were also performed using Freesurfer’s “mri_glmfit,” setting the significance at the cluster level to CWP < 0.05/3 to account for all three pair comparisons. Moreover, using “*mri_glmfit”* and the above-described strategy for the correction of *p*-values, we performed a regression analysis using each morphometric measure as the dependent variable and Vitamin D status (a continuous variable) as the independent variable of interest, also controlling for age, gender, ethnicity/race, and study site. The statistically significant clusters resulting from these analyses were then labeled after the region of the Destrieux’s anatomical atlas of sulci and gyri (Freesurfer’s aparc.2009s) (Destrieux et al., 2010) containing the maximum T-statistics.

### Other statistical analyses

In all cases, age, race sex, and study site were used as covariates in the analyses. To examine the relationships between Vitamin D groups and pain intensity/pain interference, a univariate ANOVA was employed. Partial correlation analyses were also conducted to look for associations of Vitamin D as a continuous variable with pain intensity and pain interference. Finally, linear regression analyses were employed to examine whether or not pain intensity and interference was predicted by Vitamin D status. A significance threshold of *p* < 0.05 was used for all cases.

## Results

### Participants

Across the entire sample, participants were more likely to be female (63.6%) and non-Hispanic White (NHW, 52.1%). The average age of the entire sample was 58.41 (±8.00) years. The average serum Vitamin D levels of the entire sample was 26.54 (±13.44) ng/mL. Those with optimal levels of Vitamin D tended to be female (68.2%) and NHW (54.5%), those with insufficient levels tended to be female (65.1%) and NHW (65.1%), and those with deficient levels tended to be female (58.5%) and non-Hispanic Black (NHB, 60.4%). A full breakdown of participant demographic characteristics stratified by Vitamin D level groups can be seen in Table 1. There was no significant association between BMI and Vitamin D level within the sample.

**TABLE 1:** Characteristics of the sample by vitamin D status groups.

	Total Sample	Deficient	Insufficient	Optimal	*P*-value
*Total (n%)*	140	53 (37.9)	43 (30.7)	44 (31.4%)	–
*Age*	58.41 (±8.00)	56.30 (±7.30)	60.12 (±8.55)	59.30 (±7.89)	0.005*
*Sex (n%)* *Male* *Female*	51 (36.4) 89 (63.6)	22 (41.5) 31 (58.5)	15 (34.9) 28 (65.1)	14 (31.8) 30 (68.2)	–
*Race (n%)* *NHB* *NHW*	67 (47.9) 73 (52.1)	32 (60.4) 21 (39.6)	15 (34.9) 28 (65.1)	20 (45.5) 24 (54.5)	–
*Vitamin D (ng/mL)*	26.54 (±13.44)	14.52 (±3.47)	25.14 (±3.11)	42.38 (±11.07)	–
*Depression Score (PROMIS)*	12.27 (5.67)	12.57 (5.4)	13.2 (6.3)	11.0 (3.34)	0.06
*Anxiety Score (PROMIS)*	13.06 (5.72)	13.13 (5.7)	13.7 (5.7)	12.3 (4.8)	0.306
*Pain Intensity (GCPS)*	41.9 (29.5)	49.1(±30.0)	39.7 (±24.0)	36.1 (±28.1)	0.037*
*Pain Interference (GCPS)*	32.7 (32.4)	41.4 (±34.0)	28.3 (±27.1)	27.1 (±31.8)	0.017*

*p < 0.05.

### Differences in pain intensity and interference between vitamin D groups

Mean (±SD) pain intensity ratings from the GCPS were as follows: Deficient – 49.1(±30.0); Insufficient – 39.7 (±24.0); and Optimal – 36.1 (±28.1). Mean (±SD) pain intensity ratings from the GCPS were as follows: Deficient –41.4 (±34.0); Insufficient – 28.3 (±27.1); and Optimal – 27.1 (±31.8). There was a significant effect of Vitamin D groups on pain intensity (*F*(2,140) = 3.350, *p* = 0.037). There was a significant effect of vitamin D groups on pain intensity *post-hoc* Bonferroni analyses revealed significant differences between deficient and optimal Vitamin D status (*p* = 0.044), and no statistically significant differences between optimal and insufficient as well as deficient and insufficient (*p* > 0.05) on pain intensity. There was a significant effect of Vitamin D groups on pain interference (*F*(2,140) = 4.153, *p* = 0.017). *Post-hoc* Bonferroni analyses revealed significant differences between deficient and optimal Vitamin D groups (*p* = 0.033), and deficient and insufficient Vitamin D groups (*p* = 0.051). No significant differences were noted between insufficient and optimal Vitamin D groups (*p* = 0.05).

### Continuous associations of vitamin D with pain intensity and interference

Partial correlation analyses controlling for covariates showed small, yet significant negative associations of Vitamin D with both pain intensity (*r* = 0.156, *p* = 0.031) and pain interference (*r* = 0.178, *p* = 0.014), whereby higher levels of serum vitamin D were associated with lower pain intensity and pain interference scores. In the linear regression models with covariates added, Vitamin D status significantly predicted both pain intensity (*r*^2^ = 0.024, *F*(1,140) = 4.699, *p* = 0.031) and pain interference (*r*^2^ = 0.032, *F*(1,140) = 6.615, *p* = 0.014), with higher Vitamin D scores predicting more favorable pain intensity and interference scores.

### Differences in brain volume across vitamin D levels

**FIGURE 1:**
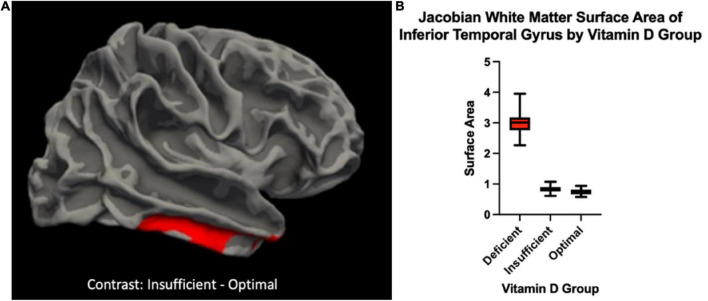
**(A)** The Jacobian white matter surface area in a cortical cluster overlapping with the right inferior temporal gyrus {region of the Destrieux atlas [FreeSurfer aparc.a2009s (Destrieux et al., 2010)] containing the maximum T-statistics} was higher for individuals with insufficient Vitamin D levels compared to those with optimal levels. To ease visual interpretation, we have used red to represent the significant positive “Insufficient – Optimal” contrast. **(B)** Box-plots depicting the distribution of the Jacobian white in the cortical vertex where the T-statistics of this group comparison was maximum for each Vitamin D level group.

**FIGURE 2:**
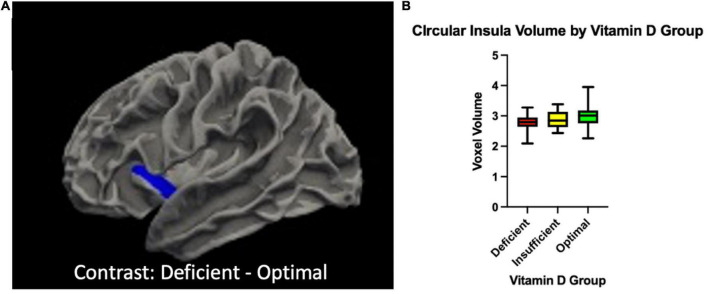
**(A)** The volume of in a cortical cluster overlapping with inferior segment of the left circular sulcus of the insula {region of the Destrieux atlas [FreeSurfer aparc.a2009s (Destrieux et al., 2010)] containing the maximum T-statistics} was higher for individuals with deficient Vitamin D levels compared to those with optimal levels. To ease visual interpretation, we have used blue to represent the significant positive “Deficient – Optimal” contrast. **(B)** Box-plots depicting the distribution of the cortical thickness in the cortical vertex where the T-statistics of this group comparison was maximum for each Vitamin D level group.

**FIGURE 3:**
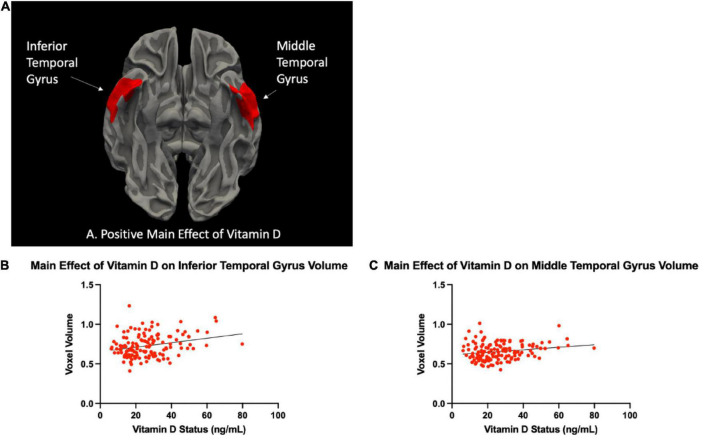
**(A)** The area of two cortical clusters overlapping with the left inferior and right middle temporal gyri {regions of the Destrieux atlas [FreeSurfer aparc.a2009s (Destrieux et al., 2010)] containing the maximum T-statistics} was higher for individuals with higher Vitamin D status. To ease visual interpretation, we have used red to represent the significant positive Vitamin D status-area correlation. Scatter plots of the cortical area in the cortical vertices where the T-statistics of these regressions were maximum, versus each Vitamin D status, are shown in panels **(B,C)**.

There were significant differences in brain volume and white matter surface area between Vitamin D level groups. Individuals classified as having optimal Vitamin D levels had greater gray matter in the left inferior segment of the circular sulcus of the insula compared to those classified as deficient (*p* = 0.045, corrected), and trended toward significance between optimal and insufficient (*p* = 0.072, corrected). There was also a positive main effect that trended toward significance of Vitamin D status group on gray matter volume of the left inferior segment of the circular sulcus of the insula (*p* = 0.054, corrected). Those with optimal levels of Vitamin D also had significantly greater surface area of white matter in the right inferior temporal sulcus compared to those with insufficient levels of Vitamin D (*p* < 0.003, corrected). When analyzing the levels of Vitamin D as continuous data, there was a significant positive main effect of Vitamin D on gray matter volume in the left inferior temporal gyrus (T3) (*p* = 0.006, corrected) and the right middle temporal gyrus (T2) (*p* = 0.012, corrected). Permutation simulations were run on all analyses. Regions and *p*-values that survived after correcting for multiple comparisons (*p* < 0.05/3 = 0.017) can be seen in Table 2. There were no significant differences in cortical thickness between groups. Brain regions that showed significant volumetric differences by Vitamin D status can be seen in Figures 1A, 2A, main effects of Vitamin D can be seen in Figure 3A, and graphical representations can be seen in Figures 1B, 2B, 3B,C.

**TABLE 2:** Significant group-level differences in brain morphometric measures among vitamin D levels.

*Group Comparison (contrast)*	Cortical morphometric measure	Hemisphere	Region containing maximum T-statistics	Maximum T-statistic	Size of Cortical Cluster (mm^2^)	MNI			CWP
						X	Y	Z	
*Insufficient – Optimal*	Jacobian white	Right	Inferior Temporal Sulcus	3.44	1272.2	53.3	−38.7	−18.3	<0.001
*Deficient – Optimal*	Thickness	Left	Inferior segment of the circular sulcus of the insula	−4.00	477.3	−38.4	−12.7	−12.6	0.015
*Main Effect of Vitamin D (regression)*	Area	Left	Inferior Temporal Gyrus	3.69	1328.1	−48.9	−12.3	−34	0.002
	Area	Right	Middle Temporal Gyrus	3.13	1185.7	53.5	−7.1	−27.9	0.004

The table shows the FreeSurfer outputs that survived Bonferroni correction across all three pairs of Vitamin D groups, i.e., CWP × 3 < 0.05). CWP values are already Bonferroni corrected across hemispheres. MNI X, Y, and Z are the coordinates in MNI space where the T-statistics was maximum. MNI, Montreal Neurological Institute. CWP, Cluster-wise P-value.

## Discussion

In our sample, middle-to-older aged individuals with knee pain, serum Vitamin D levels were associated with insular volume and white matter surface area. Specifically, we observed greater volumes of gray matter in the left inferior segment of the circular sulcus of the insula in individuals with optimal levels of vitamin D (>30 ng/mL) compared to those with insufficient (20–20.99 ng/mL) and deficient (<19.99 ng/mL) (Figures 2A,B). We also observed a larger Jacobian White Matter surface area in the inferior temporal gyrus in those with insufficient compared to optimal levels of Vitamin D (Figures 1A,B). Additionally, we also observed a positive main effect of Vitamin D level in the left inferior and right middle temporal gyri (Figures 3A–C). In general, the greatest atrophy was seen in the deficient group, followed by the insufficient and optimal groups, respectively. The neuroanatomical correlates were significant and independent of depression and anxiety, ethnicity/race, age, sex or study site in our particular sample, despite the literature noting significant differences within these groups in pain prevalence [females (Sorge and Strath, 2018) and NHBs (Strath and Sorge, 2022) disproportionately carry the burden of chronic pain] and Vitamin D status (NHBs are more likely to be deficient compared to NHWs) in previous studies (Parva et al., 2018). Moreover, Vitamin D, as well as changes in the insula and white matter tracts have previously been independently associated to chronic pain in those with these types of conditions (Martin and Reid, 2017; Cruz-Almeida and Cole, 2020). In our sample, small, yet significant correlations and regressions appeared when examining pain intensity and pain interference with vitamin D status. Thus, it is possible that potential volumetric changes in these brain regions in relation to vitamin D could be, in part, a contributor to the pain experienced by this population.

While there are many regions of the brain that have shown associations with the pain experience, one of the primary areas of interest is the insular cortex. Also known as the insula or the “Island of Reil,” is a brain region located deep within the lateral sulcus not visible to surface view. It has multiple sulci, gyri, and subdivisions, which have the potential to work independently and interactively (Augustine, 1996). While it is one of the most poorly understood regions of the brain (mainly due to its location), there is a large body of evidence suggesting that the insula is a hub that serves to coordinate a variety of information inputs across many cognitive domains and processes. The insula processes information related to speech, decision making, attention and salience processing, emotions and empathy, vestibular, chemosensory and auditory functions, and most interestingly in terms of this study – visceral sensations, somatic processes and pain (Uddin et al., 2017). An increasing number of neuroimaging and electrophysiological findings suggest that the insular cortex participates in both the sensory and affective aspects of pain - that is, the discrimination of a noxious stimulus from other stimuli or ascending/”bottom-up” modulation, and the subsequent descending/“top-down” modulation and response to noxious stimuli (Lu et al., 2016). Because the insula likely interprets and integrates information from a variety of other inputs, it is thought that it is likely serving as an interface where the pain experience is shaped in the brain. In neuroimaging studies, it has been documented that insular activation occurs regardless of noxious modality (i.e., mechanical, heat, cold, pressure, ischemia, etc.) or body part in which the stimuli were delivered (Mazzola et al., 2009; zu Eulenburg et al., 2013). Meta-analyses have suggested that clinical populations with chronic pain (such as KOA), exhibit abnormal activation patterns and disruption of networks within the insular cortex. These studies have associated these changes to abnormally atrophied gray and white matter within and surrounding the insula, relating to the reduced gray matter volume seen in this study in those with lower levels of Vitamin D.

One of the other brain regions with changes associated with Vitamin D status in our sample was the white matter of the inferior temporal sulcus. Additionally, there was a significant main effect of Vitamin D status on volume of the inferior temporal gyrus (ITG) and middle temporal gyrus (MTG). The ITG is the most ventral of the three gyri located on the lateral surface of the temporal lobe. Both the MTG and ITG have been implicated in a variety of brain processes, including visual perception and multimodal sensory integration, as well as pain processing (Onitsuka et al., 2004). In functional neuroimaging studies, evidence shows that the ITG decreases activation upon presentation with a noxious stimulus (Kong et al., 2010). Interestingly, the ITG has connections through white matter with both the anterior and posterior insular cortices (Ghaziri et al., 2017) demonstrating the potential for communication between the two regions. White matter is composed of bundles of axons that connect various brain regions into functional circuits (Fields, 2010). In the simplest sense, while gray matter facilitates information processing, white matter facilitates information transfer and allows for the communication of brain regions to one another. When the integrity of the white matter worsens and the white matter tracts begin to spread (leading to higher surface area) the neural networks and communication between brain regions is disrupted or impaired. If these connections are disrupted, sensory and motor information may not be accurately integrated into the brain and body systems (Dennis et al., 2015). We have previously shown marked decreases in white matter integrity in older adults with musculoskeletal pain, and that these decreases mediated the pain-gait relationship (Cruz-Almeida et al., 2017). White matter abnormalities have also been implicated in other pain disorders such as orofacial pain disorders (Moayedi and Hodaie, 2019) and also severity of surgical pain (Torrecillas-Martínez et al., 2020). In our study, we noted decreased surface area of white matter in the ITG in individuals with insufficient Vitamin D levels compared to those with optimal vitamin D levels. It is possible that this surface area difference could be contributing to disrupted neurological communication between brain regions, particularly ones that process pain, such as the insula and ITG.

Vitamin D (inactive: 25-hydroxyvitamin D; active: 1,25-hydroxyvitamin D) is a fat-soluble essential micronutrient that is involved in a variety of processes related to homeostasis of the human body. While it is typically known for its roles in bone health and calcium absorption, it is becoming very apparent that Vitamin D exerts influence beyond the musculoskeletal system. The discovery of the Vitamin D receptor (VDR), which is present on many cell surfaces and is necessary for successful transcription of a variety of genes (Dowd and MacDonald, 2013) – provides a pathway Vitamin D to be involved in many other bodily systems, such as the immune system and the nervous system. Recently, we and others have found associations between Vitamin D status and pain severity in various chronic pain conditions (Strath et al., 2022). However, the question of how Vitamin D is impacting the pain experience is still being answered.

Here, we found that Vitamin D levels less than 30 ng/mL were associated with less gray matter volume in the insular cortex as well as white matter surface area in the ITG region. Given that Vitamin D is a fat soluble vitamin, and that much of the brain is made up of a variety of lipids, it makes sense for Vitamin D to potentially be absorbed, used, and function in brain health (Jové et al., 2019). Studies have shown that in early life, Vitamin D is essential for brain development – many learning and memory problems, and altered neural expression of genes involved in dopamine and glucocorticoid related pathways have been found to be related to a lack of Vitamin D in childhood (Yates et al., 2018). Other studies have shown that both pre- and postnatal Vitamin D is important for optimal emotional development in children, and aids in the prevention of mental illness later on into adulthood (Freedman et al., 2018). Multiple studies also show that Vitamin D is strongly associated with preserving neurological development, protecting the brain from injury, and maintaining cognition during aging (Di Somma et al., 2017; Lau et al., 2017). Taken together, it appears that Vitamin D may play a role in the structure and function of the brain, particularly when it comes to maintaining the integrity of gray and white matter. The VDR has also been found within the central nervous system, and modulates the biosynthesis of neurotransmitters and growth factors essential for optimal brain health and development. Vitamin D has antioxidant and anti-inflammatory processes, and deficiencies have been implicated in B-amyloid deposition and loss of gray matter integrity in Alzheimer’s disease (Annweiler, 2016). Thus, it is possible that Vitamin D, or lack thereof, may be influencing the structure and function of brain regions involved in pain sensing and processing and subsequent disease outcomes. In our sample, though the coefficients were small, we noted that greater pain severity and interference was related to lower serum vitamin D, and that vitamin D status was predictive of pain intensity and interference whereby higher serum vitamin D levels were predictive of more favorable pain outcomes in both cases. As stated, the coefficients were small yet significant, likely due to the fact that there are many distinct biopsychosocial factors implicated in the pain experience, of which vitamin D status may play a role. Additionally, sociodemographic factors such as race have overlapping relationships with both vitamin D and pain severity and interference. As such, future studies evolving from the present manuscript should look at overlapping variables such as these in order to glean a clearer picture into the relationship between vitamin D and pain outcomes.

We acknowledge that there are limitations to this study. First, this was a within-subjects study design, and we do not have a pain-free control group for comparison of the results. Future studies will involve the inclusion of and analyses between pain and control groups. Second, for a smoothing kernel of 10 mm, and measures other than the cortical thickness, the threshold *p*-value of 0.01 at the voxel level could lead to ∼ 3 times more false positives than expected when using a cluster-wise non-parametric approach (Greve and Fischl, 2018) with FWE significance of 0.05. Therefore, out of the four significant result shown in Table 2, the Deficient versus Optimal comparison should not be interpreted as marginally significant, inviting to the design of future studies involving more powered samples on which our scientific questions could be tested. We did not ask about pain duration, which should be examined and accounted for in future studies, and given that this study was cross-sectional in nature we cannot infer causal relationships from these data. Future studies will take into consideration variables known to be related vitamin D status and pain outcomes such as race, as such analyses were not within the scope of this specific manuscript.

Chronic pain disorders, such as knee pain, are extremely prevalent in Western society. Pain is extremely costly monetarily and substantially impacts the well-being of the individual. If we are to make an impact on this vulnerable population, it is important that we take a holistic view of the potential mechanisms underlying pain, including nutrient status. As previously stated, Vitamin D is an important and highly utilized micronutrient, and its status has been associated with pain severity and interference outcomes across many different painful conditions. Here, we present evidence that suggests that Vitamin D status is associated with areas of the brain that participate in the sensing, interpreting, and modulation of pain. It is possible that Vitamin D deficiencies may be causing brain atrophy and functional deficits leading to exacerbated pain outcomes. Future studies looking at causal relationships between Vitamin D and brain health, as well as interventions in painful disease states are strongly encouraged.

## Data availability statement

The raw data supporting the conclusions of this article will be made available by the authors, without undue reservation.

## Ethics statement

The studies involving human participants were reviewed and approved by the University of Florida Institutional Review Board. The patients/participants provided their written informed consent to participate in this study.

## Author contributions

LS analyzed statistics and drafted the manuscript. PH and CN analyzed and interpreted neuroimaging data and revised the manuscript critically for important intellectual content. JE analyzed vitamin D samples and revised the manuscript critically for important intellectual content. LS, AJ, RF, and YC-A made substantial contributions to manuscript conception, data acquisition, analyses, and interpretation and revised the manuscript critically for important intellectual content. RF and YC-A acquired financial support for the project leading to this publication. All authors contributed to the article and approved the submitted version.

## Footnotes

1
https://github.com/Deep-MI/qatools-python
